# Chronic Myeloid Leukemia Patients Sensitive and Resistant to Imatinib Treatment Show Different Metabolic Responses

**DOI:** 10.1371/journal.pone.0013186

**Published:** 2010-10-08

**Authors:** Jiye A, Sixuan Qian, Guangji Wang, Bei Yan, Sujiang Zhang, Qing Huang, Lingna Ni, Weibin Zha, Linsheng Liu, Bei Cao, Ming Hong, Hanxin Wu, Hua Lu, Jian Shi, Mengjie Li, Jianyong Li

**Affiliations:** 1 Key Laboratory of Drug Metabolism and Pharmacokinetics, China Pharmaceutical University, Nanjing, China; 2 Department of Hematology, The First Affiliated Hospital, Nanjing Medical University, Nanjing, China; Dresden University of Technology, Germany

## Abstract

The BCR-ABL tyrosine kinase inhibitor imatinib is highly effective for chronic myeloid leukemia (CML). However, some patients gradually develop resistance to imatinib, resulting in therapeutic failure. Metabonomic and genomic profiling of patients' responses to drug interventions can provide novel information about the *in vivo* metabolism of low-molecular-weight compounds and extend our insight into the mechanism of drug resistance. Based on a multi-platform of high-throughput metabonomics, SNP array analysis, karyotype and mutation, the metabolic phenotypes and genomic polymorphisms of CML patients and their diverse responses to imatinib were characterized. The untreated CML patients (UCML) showed different metabolic patterns from those of healthy controls, and the discriminatory metabolites suggested the perturbed metabolism of the urea cycle, tricarboxylic acid cycle, lipid metabolism, and amino acid turnover in UCML. After imatinib treatment, patients sensitive to imatinib (SCML) and patients resistant to imatinib (RCML) had similar metabolic phenotypes to those of healthy controls and UCML, respectively. SCML showed a significant metabolic response to imatinib, with marked restoration of the perturbed metabolism. Most of the metabolites characterizing CML were adjusted to normal levels, including the intermediates of the urea cycle and tricarboxylic acid cycle (TCA). In contrast, neither cytogenetic nor metabonomic analysis indicated any positive response to imatinib in RCML. We report for the first time the associated genetic and metabonomic responses of CML patients to imatinib and show that the perturbed *in vivo* metabolism of UCML is independent of imatinib treatment in resistant patients. Thus, metabonomics can potentially characterize patients' sensitivity or resistance to drug intervention.

## Introduction

In the postgenomic era, the complementary use of high-throughput analytical technologies (such as genomics, proteomics, and metabonomics) in biological systems has revolutionized biological investigations. Genomic variation appears to be an important factor that can enhance or reduce the risk of developing a disease, depending on the specific genetic or epigenetic pathway involved [1], [2]. It has been demonstrated that chronic myeloid leukemia (CML) involves a translocation between chromosomes 9 and 22, which results in the expression of the BCR–ABL fusion protein. The tyrosine kinase activity of oncogenic ABL proteins is known to be essential for its transforming activity [3]. Imatinib mesylate (imatinib) is a small molecular inhibitor of the tyrosine kinase activity of the BCR–ABL fusion protein, and is now a frontline therapy for CML [4]. Despite imatinib's striking efficacy, resistance develops over time in many patients, and is more common in patients with advanced-stage CML [5]. Routine cytogenetic analysis and molecular methodologies can identify resistance or sensitivity to imatinib in CML patients, and are considered the gold standards for evaluating the potential response to imatinib in clinical practice [6], [7]. However, the two methods do not provide further molecular information about the *in vivo* metabolic perturbation involved, which may clarify the mechanism of resistance, or allow us to metabonomically characterize sensitive CML patients (SCML) and resistant CML patients (RCML). Our understanding of the biological functions involved would benefit greatly from an understanding of the metabolic network, including quantitative measurements of different types of compounds (such as proteins and metabolites) and various biochemical processes (such as gene expression) made in parallel, and preferably combined with other classical phenotypic analyses [8]. Although many researchers have monitored the response to imatinib in CML patients using molecular methodologies and cytogenetic techniques [7], [9], [10], no comprehensive metabonomic investigation has been made of the responses of CML patients to imatinib.

Metabonomics is defined as the quantitative measurement of endogenous low-molecular-weight compounds that reflect the metabolic responses of living systems to diverse stimuli [2], [11], [12]. The metabolic phenotype constitutes the endpoint of various metabolic responses and is influenced by genomic and proteomic factors. It can be used to identify early signals/biomarkers of cellular abnormalities that occur before the appearance of gross phenotypic changes [1]. Metabonomics can be used as a complementary tool, providing information about the metabolic network that cannot be obtained directly from the genotype, gene expression profiles, or even the proteome of an individual [2]. It has been successfully applied to biomedical sciences [2], [11], [13]–[9], and shows promising applications in the exploration of diseases and in the development of personalized drug treatments [2], [18], [19]. Metabonomics can potentially be applied to the discovery of tumor metabolic pathways, the investigation of metabolic responses to treatments [20], and the identification of tumor biomarkers of these responses [11], [12], [15]. In this study, using a metabonomic platform we developed based on gas chromatography/time-of-flight mass spectrometry (GC/TOFMS) and data analysis techniques [21], [22], we integrated metabonomic data with cytogenetic and molecular analyses to profile the metabolic phenotypes of CML patients and differentiate their metabolic responses to imatinib.

## Results

### CML patients

Fifty-nine patients were enrolled in the study: 53 with chronic-phase (CP) CML and 6 with blast crisis (BC). Two BC patients had a complex karyotype. Of these 59 patients, 26 were untreated (UCML). The other 33 CML patients were treated with imatinib at daily doses of 300–800 mg, and 19 achieved complete cytogenetic remission (0% Philadelphia chromosome; “sensitive CML”, SCML), whereas 14 patients were resistant to imatinib (RCML; Table 1). Seven of the RCML patients displayed primary RCML and seven displayed secondary RCML. Chronic-phase, blast crisis, and primary cytogenetic resistance were determined as described in a recent study [5]. The healthy volunteers included nine men and nine women, with a median age of 35.2 years.

**Table 1:** Information of the CML patients and the health volunteers.

Characteristics		HC	UCML	SCML	RCML
Sex	Male	9	17	17	8
	Female	9	9	2	6
Age (year)	Mean±SD	35.2±9.2	37.6±13.4	38.1±15.7	39.2±14.1
	range	18–55	18–67	23–70	18–69
Recent medicine		No	Hydroxycarbamide	Imatinib	Imatinib
Duration of disease	Mean±SD		8.3±12.6	17.8±24.2	48.5±31.6
(months)	range		1–33	6–84	6–168
Duration of imatinib	Mean±SD			12.2±23.3	25.9±12.7
treatment (months)	range			6–72	6–39
Daily dose (mg)	Mean±SD			418±52	421±77
	range			400–600	300–800

### Mutation of the ABL kinase domain in RCML patients

In the 14 resistant patients analyzed, ABL kinase domain mutations were detected in only 1 BC patient, who presented with three types of amino acid changes: L232P, F336L, and C349R.

### High-density single nucleotide polymorphism (SNP) array analysis of CML patients

After exclusion of genomic copy number polymorphisms by comparison of the data with recorded copy number polymorphisms in the UCSC Genome Browser (http://genome.ucsc.edu/) databases,a total of 44 deletions, 2 duplication, and 7 regions of loss of heterozygosity (LOH) were identified by SNP array analysis in 9 CML samples, i.e., 1 SCML, 4 primary RCML and 4 secondary RCML (Table 2). In addition to sex chromosome, four of 6 CP RCML patients did not show other abnormal genome. Moreover, Cryptic deletions on chromosome X and Y were found in patients with CP-CML both in SCML and RCML.

**Table 2:** Lesion detected in CML samples by SNP array analysis.

Patients ID	Chromosome	Genic status	Starting position	Ending position	Genes identified	Disease status	Response to imatinib
R01C02	5	Del	81629356	115815122	LOC92270	BC	Resistance
R01C02	9	Del	127196469	132665560	FREQ,CDK9	BC	Resistance
R01C02	22	Del	22053721	22902745	GSTTP1, LOC388882, IGLL1, C22orf16, MMP11, SLC2A11, MIF, FLJ31568	BC	Resistance
R01C02	22	Dup	23995985	24240879	LRP5L, ADRBK2	BC	Resistance
R06C01	7	Del	61862616	61921953	No gene	CP	Resistance
R06C01	19	Dup	32493654	33528102	LOC727780,LOC642290,LOC727771	CP	Resistance
R02C01	11	Del	50354779	51228612	OR4A5, LOC646813	CP	Resistance
R02C01	19	Del	20423788	20514068	LOC645490	CP	Resistance
R04C01	11	Del	46327343	47374448	F2,SPI1,DGKZ,AMBRA1,MADD C11orf49,PACSIN3	BC	Resistance
R04C01	17	Del	44176361	52843822	>20 genes	BC	Resistance
R04C01	2	LOH	78822450	89733531	>20 genes	BC	Resistance
R04C01	2	LOH	98364441	109797238	>20 genes	BC	Resistance
R04C01	2	LOH	109809150	121111631	>20 genes	BC	Resistance
R04C01	3	LOH	142389922	153466638	>20 genes	BC	Resistance
R04C01	5	LOH	156922335	168529461	>20 genes	BC	Resistance
R04C01	15	LOH	82248349	100215359	>20 genes	BC	Resistance
R04C01	20	LOH	29310063	55040991	>20 genes	BC	Resistance
R03C02	1	Del	240744201	241169492	LOC391183, PLD5	CP	Sensitivity

Starting position: start position of genomic lesion; Ending position: end position of genomic lesion;LOH: loss of heterozygosity, Del; deletion, Dup; duplication.BC,blast crisis; CP, chronic-phase.

### Acquired GC/TOFMS data and the identification of compounds

**Figure 1:**
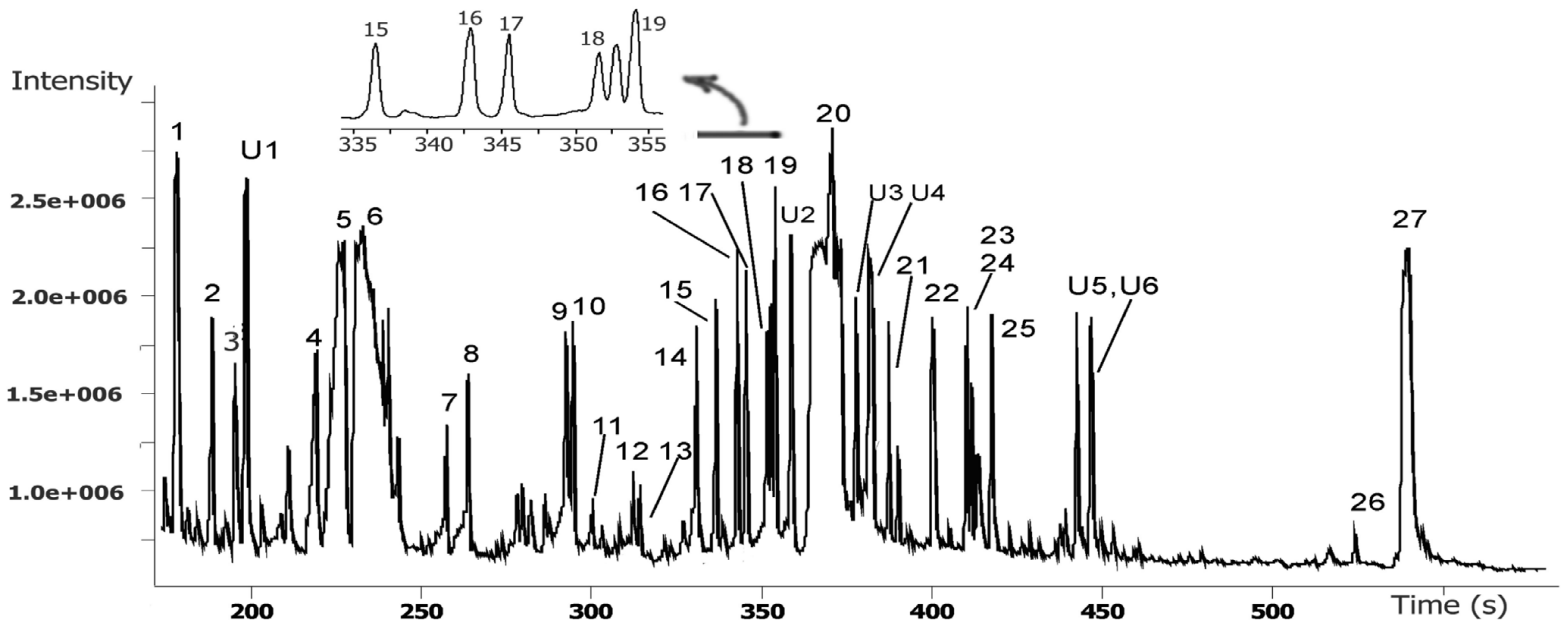
A typical GC/TOFMS chromatogram of blood plasma. Several hundreds of peaks can be detected at only one analysis and some of the peaks were identified. Part of the chromatogram was zoomed in for clear inspection of the peaks, 15–19, retention time from 335 to 355 s. 1, Lactate; 2, butyamine; 3, 3-hydroxybutyrate; 4, valine; 5, urea; 6, phosphate; 7, serine; 8, threonine; 9, pyroglutamate; 10, hydroxyproline; 11, creatinine; 12, ornithine; 13, phenylalanine; 14, methyl myristate (QC reference standard); 15, glutamine; 16, glycerol-3-phosphate; 17, azelate; 18, citrate; 19, [^13^C_2_]-myristic acid (stable isotope internal standard); 20, glucose; 21, palmitic acid; 22, uric acid; 23, linoleic acid; 24, oleic acid; 25, stearic acid; 26, alpha-tocopherol; 27, cholesterol; U1-5, unidentified peaks of retention indices at 1163, 1876, 1986, 2015, 2415, 2448, respectively.

A typical total-ion-current chromatogram showed several hundred peaks in a single analysis (Figure 1). After deconvolution of the chromatograms, much quantitative and qualitative information was obtained. In general, a total of 186 non-targeted peaks/metabolites were detected, and a data matrix of 186×77 (77 observations/sample) was constructed. Altogether, 22 peaks were excluded from further data processing because they were not detected in the normal control and might have been artifacts derived from drug metabolites or other sources. In consequence, 96.97% of the peak areas were validated, with data missing for 382 peak areas (these GC/TOFMS responses are very low) in the whole data matrix of 164×77. By comparison with authentic reference standards or reference compounds in available libraries, 72 compounds were identified, including amino acids, organic acids, amines, saccharides, lipids, fatty acids, etc. (Table S1).

### Metabolic patterns of UCML, SCML, and RCML

**Figure 2:**
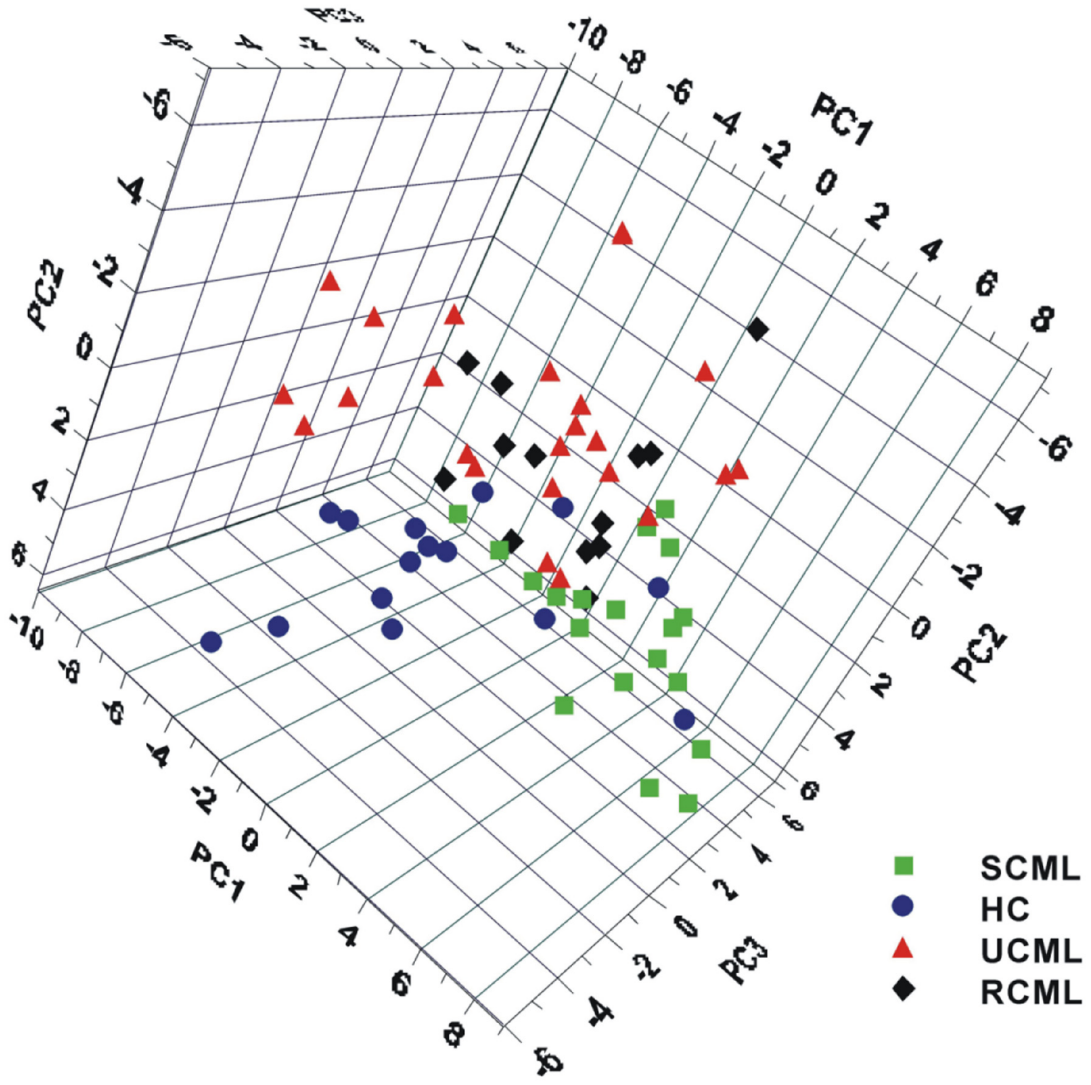
The scores plot of PLS-DA model (3 principal components) of the four groups. UCML, SCML, RCML and the healthy control (HC). This figure shows the difference between UCML and the healthy control and SCML and UCML, respectively. The overlapping of UCML and RCML suggested similar metabolic phenotype and indicated the therapeutic ineffectiveness of imatinib on RCML. The overlapping of SCML and HC suggested similar metabolic phenotype and the therapeutic effectiveness of imatinib on SCML.

For an overview of the dataset, an unsupervised principal components analysis (PCA) was applied to the data. Three serious outliers were identified, one each from the healthy control group (HC), UCML, and RCML. They were excluded from further data analysis because serious outliers negatively affect and thus bias the model. To calculate a partial least-squares projection to latent structures and discriminant analysis (PLS–DA) model, the remaining samples were classified into four groups (HC, UCML, SCML, and RCML). According to the cross-validation [23], the PLS–DA model of the three principal components explained 30.8% of the variation in X (R^2^X = 0.308, GC/TOFMS response variables/peaks) and 39.7% of the variation in Y (R^2^Y = 0.397, sample types), and predicted 23.9% of the sample types. Although the four groups overlapped to some extent on the scores plot (Figure 2), obvious separations were observed for HC, SCML, and UCML. UCML clustered far from SCML and HC, whereas RCML was scattered and primarily overlapped with UCML, suggesting that its metabonomic composition was similar to that of UCML, but different from those of HC and SCML.

A submodel was calculated to further evaluate the metabolic similarities and differences among SCML, HC, and UCML. The two-principal-components PLS–DA model (Figure S1) showed that SCML clustered far from UCML, whereas it overlapped HC, clearly suggesting that the sensitive metabolome was regulated towards the normal profile.

### Metabonomic differences between UCML patients and HC

A PLS–DA model between UCML and HC clearly identified the similarities in the two groups and the significant differences between the two groups. To better understand the intrinsic variations, specific compounds were identified. Statistical analysis (one-way analysis of variance, ANOVA) showed higher levels of glutamine, myo-inositol, arabinose, glycine, urea, ornithine, glutamate, pyroglutamate, and an unidentified compound, but lower levels of citrate, high-density lipoprotein (HDL), linoleate, and some unidentified peaks in UCML (*P*<0.05; Table 3). For the other compounds, such as pipecolate, pseudouridine, urate, α-tocopherol, adipate, total cholesterol, and nonesterified cholesterol (free form), the differences between HC and UCML were obvious but not statistically significant (*P*>0.05).

**Table 3:** The relative abundance of the identified compounds/peaks differentiating UCML from HC and the regulation by imatinib.

Variable Name	Healthy control(HC)			UCML				Fold change	SCML					RCML				
	Mean	±	SD	Mean	±	SD		UCML/HC	Mean	±	SD			Mean	±	SD		
Pipecolate	66738	±	27812	191019	±	150758		2.864	113166	±	69717			164293	±	105135	*	
Glutamine	397746	±	178241	731628	±	477666	*	1.839	642994	±	256423		*	744690	±	508088	*	
myo-Inositol	2567687	±	1211442	4224531	±	2076166	*	1.645	2460364	±	851377	††		3201257	±	2305927		
Arabinose	160888	±	54011	263240	±	134590	**	1.636	169357	±	57379	†		206307	±	106486		
Glycine	4994510	±	1934972	7755324	±	3431521	*	1.553	5697956	±	1783735			7589657	±	3502635		
Urea§ (mmol/L)	4.95	±	0.94	7.38	±	1.12	*	1.491	5.10	±	1.21			7.49	±	1.41	*	
Ornithine	7051554	±	3092520	10132171	±	3809942	*	1.437	9154095	±	4566462			10755744	±	5278905		
Pseudouridine	187540	±	93536	262470	±	132366		1.400	123979	±	55572	†		230273	±	142358		
Glutamate	486771	±	129371	672095	±	230155	*	1.381	475953	±	94505	†		591183	±	252764		
Pyroglutamate	12872691	±	3860744	17366747	±	3905589	**	1.349	14246015	±	3677287	†		16553496	±	3610300	*	
Urate	8635673	±	5325414	9311846	±	5403118		1.078	11613451	±	3705793			9157485	±	3361998		
CPU_ DB5_RI1341_P	66738	±	27812	191019	±	99008	*	2.862	113166	±	69717			164293	±	105135		
Citrate	9277095	±	2814476	7138455	±	2012240	*	−1.300	8736984	±	1696040			7197272	±	2641511		
alpha-Tocopherol	808681	±	165795	601033	±	195359		−1.345	683782	±	282667			535638	±	240045	*	
Linoleate	8552168	±	3885328	5109234	±	2596822	*	−1.400	6439932	±	3154482			5584365	±	3110760		
Adipate	202105	±	130022	135086	±	105583		−1.495	164819	±	69713			168152	±	82656		
HDL § (mmol/L)	1.38	±	0.33	0.83	±	0.25	**	−1.663	1.27	±	0.26	†		0.87	±	0.39		
Total cholesterol§ (mmol/L)	4.57	±	1.28	3.28	±	1.03		−1.393	3.79	±	0.60			2.76	±	0.77		
Non-esterified cholesterol	66222510	±	11227958	61596173	±	12264833		−1.075	59449993	±	10150252			55116351	±	10009437		
CPU_ DB5_RI2494_P	4252017	±	1132727	3208128	±	1021383	*	−1.325	4008764	±	1149047	†		3019455	±	826266	**	‡
CPU_ DB5_RI2664_P	988481	±	549499	648226	±	389483	*	−1.525	1080495	±	309271	†		728768	±	315890		‡
CPU_ DB5_RI2124_P	177008	±	101696	105477	±	98053		−1.678	186469	±	97861	†		110002	±	57932		

All peaks areas were normalized by the stable isotope internal standard, [^13^C_2_]-myristic acid; CPU_ DB5_RI*x*_P: Unidentified compounds in plasma samples detected with DB-5 capillary column in GC/TOFMS, China Pharmaceutical university (CPU); RI, retention index; *x*, retention time index value; P, plasma sample.

* ,**: statistically significant different from that in HC, p<0.05, 0.01;

† , ††: statistically significant different from that in UCML, p<0.05, 0.01;

‡ , ‡‡: statistically significant different from that in SCML, p<0.05, 0.01.

§ , clinic measurement results.

### Metabolic perturbation of CML after imatinib treatment

To facilitate the identification of the compounds that were affected by exposure to imatinib, three separate PLS–DA models were computed between SCML, RCML, and UCML. The first model between SCML and UCML explained 28.3% of the GC/TOFMS response variables/peak areas and 74.2% of the sample types, and also predicted 47.3% of the sample types. The relatively low capacity to explain the GC/TOFMS response variables (28.3%) means that the majority of them were not significantly affected, i.e., not many metabolites were affected in SCML. Another model between SCML and RCML resulted in a clustering similar to that of the previous model, suggesting that metabolic responses to imatinib were evident in these two groups of patients. However, the model between RCML and UCML failed to differentiate the two groups according to cross-validation, which resulted in a negative prediction parameter, Q^2^Y, of −10.2%. This means that the metabonomic composition of RCML was not significantly different from that of UCML. In summary, in terms of metabonomics, SCML patients differed from UCML, whereas RCML patients were similar to UCML patients.

Compared with UCML patients, significant reductions in myo-inositol, arabinose, pseudouridine, glutamate, and pyroglutamate, and increases in HDL and three unidentified peaks were observed in SCML patients (*P*<0.05, one-way ANOVA; Table 3). Pipecolate, glutamine, glycine, urea, ornithine, citrate, α-tocopherol, and linoleate levels were also more or less rectified, although not statistically significantly (*P*>0.05), but most of them were adjusted towards normal levels. Interestingly, none of the metabolites listed in Table 3 were significantly different in RCML and UCML patients. The results both from the general scores plot (Figure 2) and for specific compounds (Table 3) confirmed that RCML was quite similar to UCML.

### Metabonomic differences between RCML BC patients and RCML CP patients

RCML BC patients showed a totally different metabonomic phenotype from that of RCML CP patients (Figure S2). It is noteworthy that many compounds, including oleate, palmitate, linoleate, stearate, glycine, pyroglutamate, arabinose, β-d-methylglucopyranoside, and fumarate, showed significant elevation in RCML BC patients compared with those in RCML CP patients (Table S2). Lysine was the only compound occurring at a lower level in RCML BC patients.

## Discussion

Although the correlations between the cytogenetic and molecular effects and clinical efficacy have been well established, previous studies have provided little information about the *in vivo* metabolism of CML patients during imatinib treatment, except in terms of bone and mineral metabolism [24], hyperlipidemia [25], creatine kinase, phosphate, and phosphocholine [26]–[27]. Hundreds of low-molecular-weight endogenous compounds in the plasma were profiled here with a high-throughput GC/TOFMS analysis, and many metabolites that distinguished the different CML treatment groups were identified. Factors such as sex, age, background, disease duration, imatinib treatment duration, and diet can massively affect metabonomics and may muffle the variation in different groups. Despite these confounding factors, our results not only show that UCML differs from HC in terms of metabonomics, but also characterize SCML and RCML, and suggest their similarity to HC and UCML, respectively. This indicates that metabolic profiling can potentially characterize the status of CML in response to imatinib (ineffective or effective).

### Metabonomic differences between UCML patients and HC

The significant metabonomic differences between UCML and HC indicate metabolic perturbation in CML patients. Of the metabolites contributing most to the differences between UCML and HC, intermediates of the tricarboxylic acid (TCA) cycle (citrate) and lipid metabolism (cholesterol, HDL, linoleate) were downregulated in UCML, whereas urea cycle metabolites, such as pyrimidines (pseudouridine), amino acids (glycine, ornithine), and purines (urate), were elevated to some extent (Table 3, Figure S3). Consistent with the results for other tumors [16], [28], some amino acids, such as glutamate, ornithine, glycine, and pyroglutamate, were found at higher levels in UCML compared with those in HC, which indicates a cellular requirement for a higher turnover of structural proteins. Purines (urate) were detected at higher levels in UCML, indicative of a higher capacity for DNA synthesis. After the imatinib intervention, we detected a higher level of urate in SCML than in UCML, which is supported by a previous report [27]. The relatively lower level of citrate suggests the downregulation of the TCA cycle and that CML cells require higher-energy metabolism. This is achieved through the upregulation of amino acid transporters, cellular molecule synthesis, and signal transduction by the delivery of carbon backbones. Increased glycolysis has been consistently observed in cancer cells [28], [29], but whether this metabolic shift is a consequence or cause of cancer remains controversial [30].

### Metabonomic differences between SCML and RCML

After imatinib treatment, SCML patients underwent an obvious metabolic perturbation, whereas RCML patients were not significantly affected. The relevant changes in regulatory pathways, in response to the drug intervention, can be discerned from the discrete transcriptomic and metabonomic datasets [31]. Based on our metabonomic results, both the mathematical PLS–DA model and the suggested markers offer alternative diagnostic methods to cytogenetics.

To evaluate the genomic effect on metabolic phenotype, SNP array analysis was applied to identify the difference between CP & BC CML, and SCML & RCML. Our data are consistent with a recent SNP array study that submicroscopic alterations were detectable on chromosome 1,9,17 and 22 [32]. Deletions, duplication and LOH on chromosome 17, 9, 22, 5 and 19 were identified in several important chromosomal regions of BC patients. Chromosome 17 was most heavily affected by secondary genomic alterations on development of TKI resistance during disease progression [32], [33]. The deletions and amplifications on chromosomes 9 and 22, which were suggested to be associated with TKI resistance or disease progression [32], were detectable in 1 secondary resistant sample developing imatinib resistance. In contrast to the lesions on chromosome 17, 9 and 22, chromosome 1 may be involved in the initial development of CML rather than associated with TKI resistance [32], just as one of SCML patients showed deletion on chromosome 1 (Table 2).

Of the 14 RCML patients, only one BC patients carried ABL kinase domain mutations, so the different metabolic phenotypes of RCML and SCML are not necessarily dependent on ABL domain mutations. The difference is probably attributable to the activation of other pathways for the survival and proliferation of CML cells [34]. On the other hand, deletion or duplication on chromosome 1, 11 or 22, may lead to abnormal expression of genes which are all involved in cell lipid metabolism, including PLD5, LRP5L and AMBRA1. One of the genes in this deleted region on chromosome 22 was SLC2A11, a class II sugar transport facilitator, which exhibits highest similarity with the fructose transporter GLUT5. Three studies have reported expression signatures of CD34+ cells in relation to CML blast crisis and duration of chronic phase in patients treated with nonimatinib or imatinib therapy, respectively [35], [36], [37]. Comparison with both the Zheng et al.[35] and Yong et al [36] data, McWeeney et al.[37] found five genes (CSTA, RNASE3, PRTN3, PLAUR, and MPO) overlapped between the 3 datasets. Unfortunately, we failed to associate the metabolic data and the published genomic data.

The metabonomic data (Table S2) indicate that BC cells maintain a highly glycolytic status, with increased glucose and urate production, and require more source materials (such as amino acids and lipids) to support cell proliferation. Taken together, it strongly suggested that metabolic variation between BC and CP patients were closely related to genomic alterations.

Conversely, as in other solid cancer cells, the leukemia cells exhibited increased glycolysis, and took advantage of this metabolic pathway for ATP generation as a main source of energy. This phenomenon is known as the “Warburg effect” and is considered one of the most fundamental metabolic changes that occur during malignant transformation [28], [29], [38]. Imatinib reverses the Warburg effect in BCR–ABL-positive cells by switching from glycolysis to mitochondrial glucose metabolism, resulting in a reduction in glucose uptake and a higher energy state [38]. The metabolic phenotype of SCML showed modification of the TCA cycle and lipid metabolism, together with the upregulation of *de novo* nucleotide triphosphate/deoxynucleotide triphosphate synthesis [38]. In accordance with a previous study, these results suggest that imatinib exerts its effect by arresting DNA replication and the cell-proliferation cycle [11].

In this study, both HDL and total cholesterol decreased in RCML and increased in SCML. Cholesterol is an integral component of all eukaryotic cell membranes and is essential for normal cellular function. It is well known that the major function of HDL is to maintain cholesterol homeostasis in normal cells through the removal of excess cholesterol from intracellular pools. Plasma HDL concentrations reflect the activity of a number of intra- and extracellular metabolic pathways. The plasma levels of this lipoprotein can be determined by the flow of cholesterol through the extracellular pathways [39]. A reduction in HDL correlates with the accumulation of cholesterol esters during cell proliferation [40]. We presume that the low level of HDL observed in our study was associated with increasing BCR–ABL-mediated cell proliferation and a resistance to imatinib *in vivo*.

### Metabonomic differences between SCML and HC

The modification of the metabolome and the genome occurred synchronously during effective treatment with imatinib, followed by a reduction in the burden of CML cells. The metabolic phenotype of SCML was similar to that of HC, but the two groups did not completely overlap, even after years of treatment with imatinib. This result reinforces the observation that SCML patients are in fact genetically different from HC patients. We postulate three reasons why the metabolomes of SCML patients did not completely overlap those of HC. First, although the active CML cells that were sensitive to imatinib shifted towards a normal metabolism, quiescent and residual CML cells may influence the biochemical networks [41]. Second, besides its positive effect on CML, imatinib may affect other metabolic pathways. For example, Rowley proposed that imatinib treatment potentially targets the *BCR–ABL* fusion gene but may disrupt critical pathways required for normal cell function [42]. Third, many patients who were translocation negative in a standard cytogenetic analysis showed very low amounts of the BCR–ABL fusion on reverse transcriptase–polymerase chain reaction (PCR) analysis [6]. Therefore, we believe that patients with SCML, even those with a normal karyotype, still maintain a diseased status.

In summary, our results not only identify metabonomic differences between UCML and HC, but also demonstrate and correlate the genetic and metabolic responses to the imatinib intervention. Therefore, the metabonomic profiling described here may be useful for monitoring the metabolic changes that occur following imatinib treatment of CML. The analysis of metabolic changes may also be used as an alternative method of characterizing individual responses to imatinib in a population of CML patients, which may ultimately lead to personalized therapeutic strategies. This study opens the way to the identification of gene-to-metabolite networks, and offers a method for the early prediction of responsiveness to imatinib and the personalization of treatment, thus improving clinical and therapeutic decisions.

## Materials and Methods

### Chemicals

All the authentic reference standards and reagents were of analytical, silylation, or chromatography grades, and were purchased from Sigma-Aldrich (St Louis, MO, USA), Merck (Darmstadt, Germany), Fluka (Buchs, Switzerland), Aldrich (Steinhein, Germany), Serva (Heidelberg, Germany), Sigma-Aldrich (Isotec, USA), and Pierce Chemical Company (USA). Distilled water was produced with a Milli-Q Reagent Water System (Millipore, MA, USA).

### Patients and plasma samples

Fifty-nine male and female individuals attending Jiangsu Province Hospital, aged 18 years or older, were eligible for inclusion if they had been diagnosed with CML between January 2007 and September 2008. Of these patients, 26 were untreated or were treated with hydroxycarbamide, and were designated “untreated CML” (UCML), and 33 were treated with 300–800 mg/d imatinib. Eighteen healthy adult volunteers with no genetic, hepatic, renal, or cardiovascular disease were selected by matching them in age and sex to the CML patients. Diet, weight, and levels of physical activity remained unchanged during the course of the imatinib therapy. All the patients and healthy volunteers provided their written informed consent before enrollment in the study. The study was approved by the Institutional Review Board of the Nanjing Medical University.

### Cytogenetic analysis

Bone marrow was obtained from the patients. The karyotypes were evaluated with an R-banded analysis of 20 metaphase cells from unstimulated, cultured (24 h) bone-marrow aspirates. The karyotypes were described according to the international system for human cytogenetic nomenclature guidelines [43]. Chronic-phase, blast crisis, and primary cytogenetic resistance were as defined in recent studies [5], [6].

### Analysis of mutations in the ABL kinase domain

DNA was extracted from peripheral blood or bone marrow samples with a standard phenol/chloroform extraction method. The primer pairs were designed with primer3 software (http://frodo.wi.mit.edu/cgi-bin/primer3/primer3_www.cgi) to amplify the coding sequence of *ABL* (Table S3). The ABL kinase domain of the *BCR–ABL* allele was amplified with a PCR–restriction fragment length polymorphism technique, followed by direct sequencing and sequence homology analysis. Sequencing was performed with the BigDye Terminator Cycle Sequencing Ready Reaction Kit (PerkinElmer BioSystems, Foster City, CA, USA) on an ABI Prism 3700 DNA Analyzer (PerkinElmer BioSystems).

### SNP array analysis

Genomic DNA isolated from cells was subjected to GeneChip Human mapping microarray (Shanghai SNP-chip, Illumina,Inc, CA, USA). High-quality genomic DNA was processed in accordance with the genomic mapping 250K NspI protocol and hybridized to 250K NspI SNP arrays according to the manufacturer's instructions. Data analysis of deletions, amplifications and loss of heterozygosity (LOH) was carried out using the CNAG (copy number analysis for Affymetrix GeneChips)software with nonmatched references.

### Sample preparation and GC/TOFMS analysis

Plasma was isolated and treated as previously reported [22], with a few modifications. Briefly, 100 µL of plasma was thawed, 400 µL of methanol was added to the extract, and the protein was precipitated. [1,2-^13^C_2_]-Myristic acid (2 µg) was added as the internal standard. The supernatant (100 µL) was dried, methoximated, trimethylsilylated, and prepared for analysis. To extract more information from the study, triglyceride (TC), urea, total cholesterol (TG), and high- and low-density lipoproteins (HDL and LDL, respectively) were determined with an automatic biochemical analyzer (Olympus AU5400, Japan) at Nanjing Medical University.

To minimize the systematic variation, all plasma samples were randomly analyzed by GC/TOFMS using a previously reported method [22]. To achieve better chromatographic separation, the column temperature was initially maintained at 70°C for 2 min, and then increased at a rate of 35°C/min from 70°C to 305°C, where it was maintained for 2 min. Masses were scanned and the data were acquired from m/z 50–800 at a rate of 30 spectra/s.

### Data processing and pattern recognition

Automatic peak detection and mass spectrum deconvolution were performed with the ChromaTOF™ software (version 3.25) and the peak areas were obtained as reported previously [21]. A data matrix of the peak areas was thus constructed, with the observation/samples in the first column and the responses/peaks as variables in the first row. The retention index was calculated by comparing the retention times of the peaks with those of the alkane series C8–C40. The compounds were identified by the comparison of the mass spectra and retention indices of all the detected peaks with authentic reference standards available in the National Institute of Standards and Technology library 2.0 (2005) and an in-house mass spectra library database maintained by Umea Plant Sciences Center, Sweden.

Pattern recognition was performed with the professional data processing software SIMCA-P 11 (Umetrics, Umeå, Sweden). PCA and PLS–DA were used to analyze the data with a standard method [44]. The statistical results of the normalized peak areas were validated with one-way ANOVA.

## Supporting Information

Figure S1 The PLS_DA scores plot of SCML, UCML and healthy control (HC). The overlapping of SCML and HC suggested the rectification of endogenous compound in SCML towards normal. While the distinct separation of SCML and UCML suggested metabolic responses to imatinib.
(0.32 MB TIF)

Figure S2 The PLS_DA scores plot of RCML BC (▪, n = 6) and RCML CP (□, n = 8) patients. RCML BC patients showed totally different metabonomic phenotype from that of RCML CP patients.
(0.16 MB TIF)

Figure S3 Metabolic perturbations in UCML and metabolic response to imatinib treatment. The marked metabolites were observed at an abnormal level in UCML, and most of them were regulated towards normal in SCML with imatinib. Red or green blocks represent a statistically significant higher or lower level(p<0.05, one-way ANOVA) in UCML in comparison with the healthy control, respectively. Pink and blue blocks represent a higher or lower (not statistical significance, p>0.05, one-way ANOVA) level in UCML in comparison with healthy control, respectively.
(0.20 MB TIF)

Table S1 Compounds identified in human blood plasma.
(0.12 MB DOC)

Table S2 The relative abundance of the identified compounds/peaks differentiating RCML BC from RCML CP patients.
(0.06 MB DOC)

Table S3 Primer pairs used for PCR.
(0.04 MB DOC)
