# Supplementary figures and images for: Chronic Myeloid Leukemia Patients Sensitive and Resistant to Imatinib Treatment Show Different Metabolic Responses
Source: PLoS One. 2010 Oct 8;5(10):e13186. doi: 10.1371/journal.pone.0013186 (PMC2951899; doi:10.1371/journal.pone.0013186)

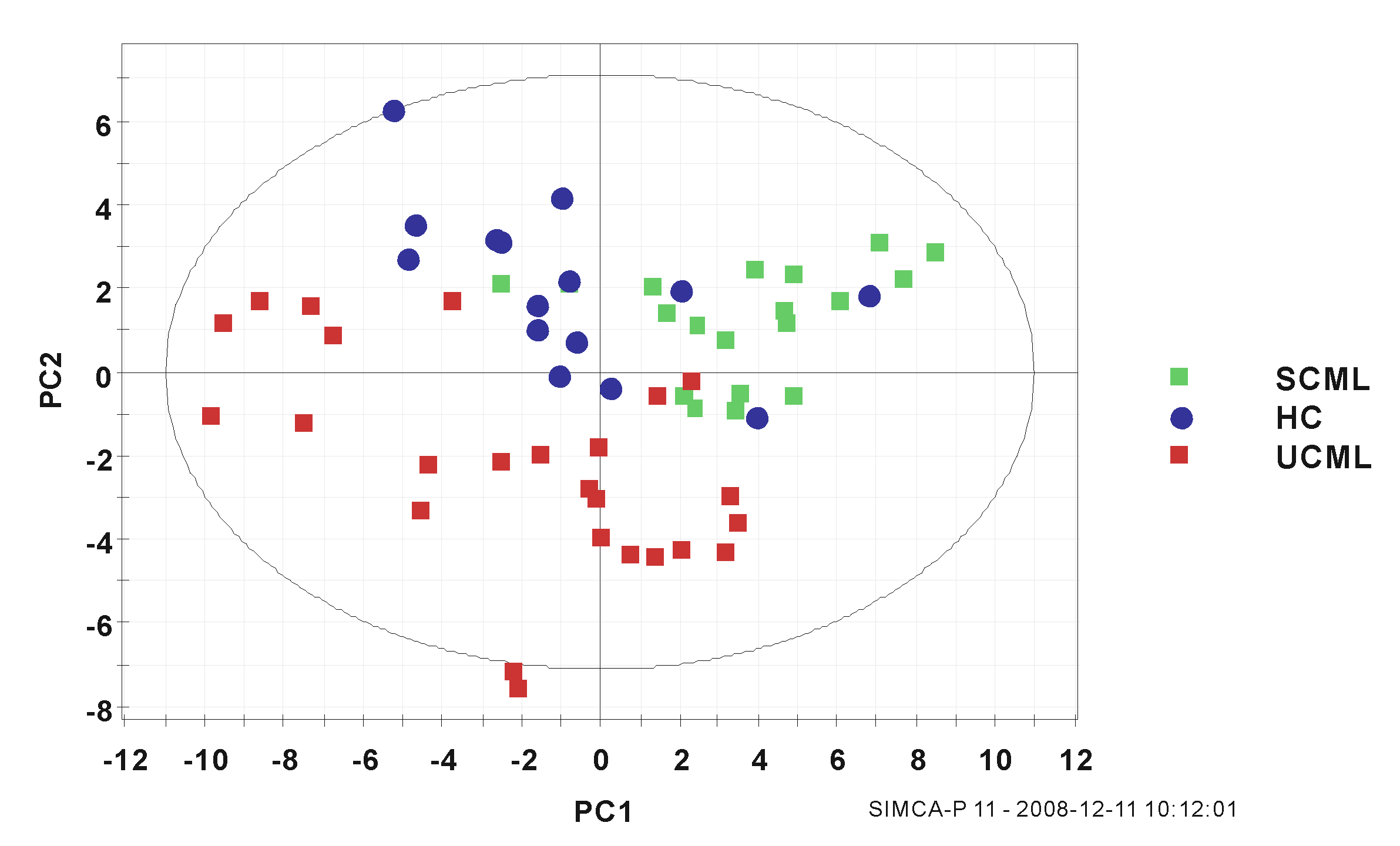

Supplement: Figure S1 — The PLS_DA scores plot of SCML, UCML and healthy control (HC). The overlapping of SCML and HC suggested the rectification of endogenous compound in SCML towards normal. While the distinct separation of SCML and UCML suggested metabolic responses to imatinib. (0.32 MB TIF) [file pone.0013186.s001.tif]

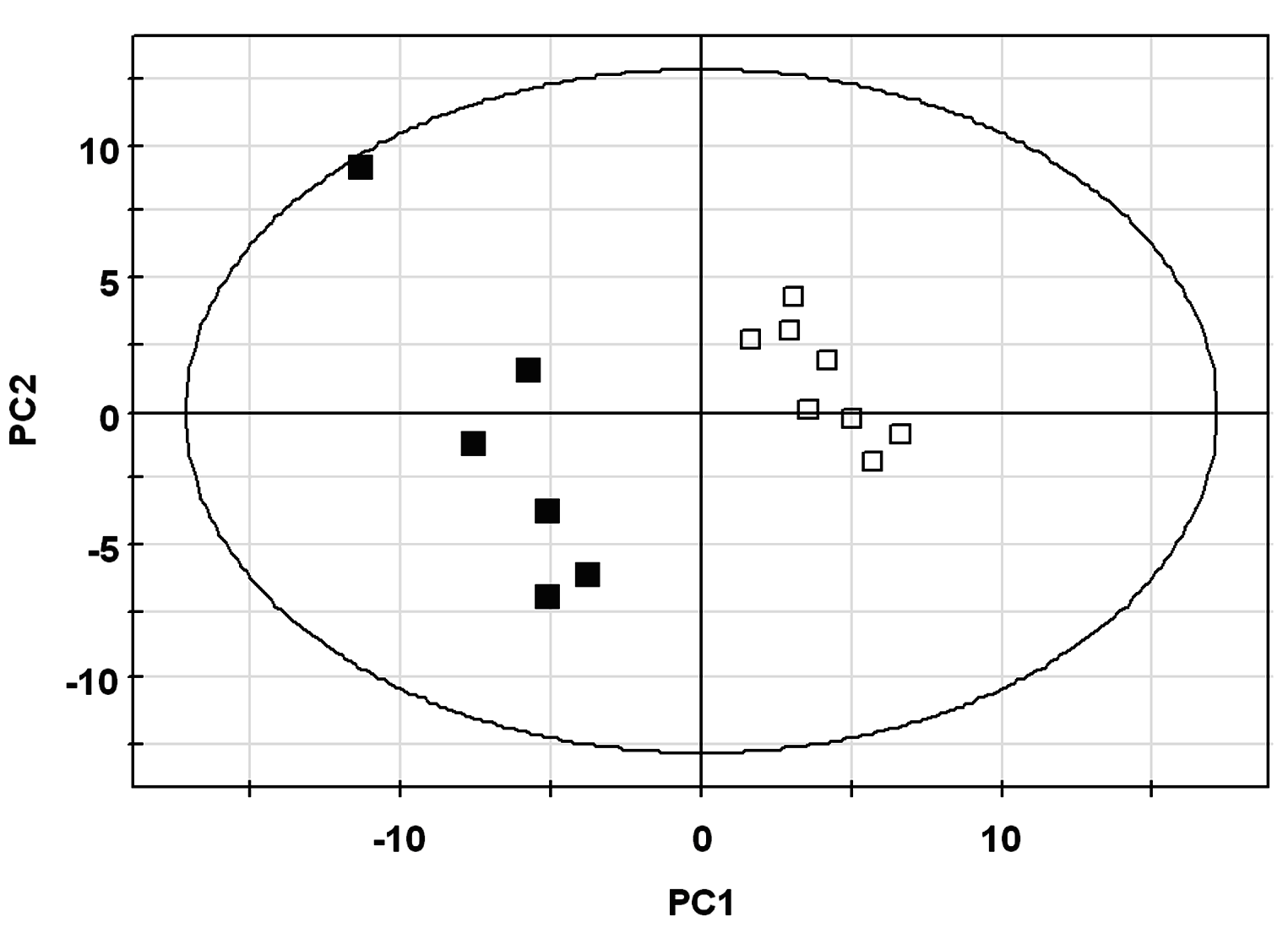

Supplement: Figure S2 — The PLS_DA scores plot of RCML BC (▪, n = 6) and RCML CP (□, n = 8) patients. RCML BC patients showed totally different metabonomic phenotype from that of RCML CP patients. (0.16 MB TIF) [file pone.0013186.s002.tif]

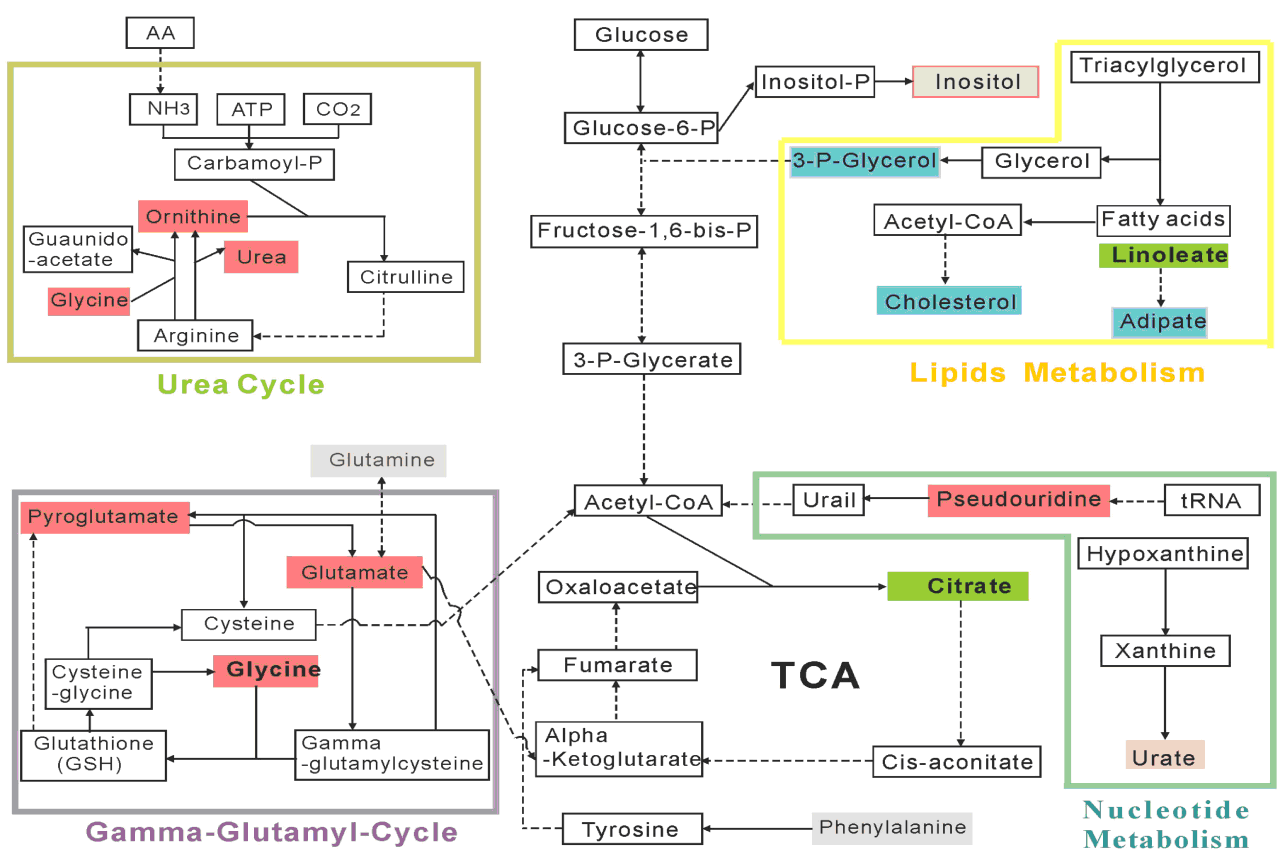

Supplement: Figure S3 — Metabolic perturbations in UCML and metabolic response to imatinib treatment. The marked metabolites were observed at an abnormal level in UCML, and most of them were regulated towards normal in SCML with imatinib. Red or green blocks represent a statistically significant higher or lower level(p<0.05, one-way ANOVA) in UCML in comparison with the healthy control, respectively. Pink and blue blocks represent a higher or lower (not statistical significance, p>0.05, one-way ANOVA) level in UCML in comparison with healthy control, respectively. (0.20 MB TIF) [file pone.0013186.s003.tif]
