# Supplementary material for: Chronic Myeloid Leukemia Patients Sensitive and Resistant to Imatinib Treatment Show Different Metabolic Responses
Source: PLoS One. 2010 Oct 8;5(10):e13186. doi: 10.1371/journal.pone.0013186 (PMC2951899; doi:10.1371/journal.pone.0013186)
Supplement: Table S1 — Compounds identified in human blood plasma. (0.12 MB DOC) [file pone.0013186.s004.doc]

**Supporting information**

**Table S1 Compounds identified in** human blood plasma

| **Identified compounds** | **Derivatives** | **Retention Index** |
| --- | --- | --- |
|
| Lactate | O-2TMS | 1063 |
| Hydroxyacetate | O-2TMS | 1078 |
| Butyamine | N-2TMS | 1106 |
| Alanine | N,O-2TMS | 1110 |
| 2-Hydroxybutyrate | O-2TMS | 1138 |
| Glycine | N,O-2TMS | 1141 |
| 3-Hydroxybutyrate | O-2TMS | 1163 |
| 2-Aminobutyric acid | N,O-2TMS | 1169 |
| Urea | N-3TMS | 1201 |
| Phosphorate | O-3TMS | 1229,1291 |
| Valine | N,O-2TMS | 1232 |
| Urea | N-2TMS | 1251 |
| Leucine | N,O-2TMS | 1284 |
| Proline | N,O-2TMS | 1308 |
| Glycine | N,O-3TMS | 1319 |
| Succinate | O-2TMS | 1323 |
| Pipecolate | O-2TMS | 1339 |
| Glycerate | O-3TMS | 1345 |
| Fumarate | O-2TMS | 1352 |
| Serine | N,O-3TMS | 1364 |
| Threonine | N,O-3TMS | 1402 |
| Glutarate | O-2TMS | 1410 |
| Methyl-cysteine | N,S-2TMS | 1429 |
| Aminomalonate | N,O-3TMS | 1481 |
| 2,5-Diaminovalerolactam | N-2TMS | 1504 |
| Adipate | O-2TMS | 1508 |
| Pyroglutamate | N,O-2TMS | 1532 |
| Hydroxyproline | N,O-3TMS | 1540 |
| Aspartate | N,O-3TMS | 1542 |
| Cysteine | N,S,O-3TMS | 1568 |
| Creatinine | N,O-3TMS | 1569 |
| Threonate | O-4TMS | 1582 |
| Alpha-ketoglutarate | MEOX, O-2TMS | 1586 |
| Glutamine | N,O-4TMS | 1602, 1749 |
| Ornithine | N,O-3TMS | 1623 |
| Glutamate | N,O-3TMS | 1628 |
| Phenylalanine | N,O-2TMS | 1637 |
| p-Hydroxyphenylacetate | O-2TMS | 1641 |
| Asparagine | N,O-3TMS | 1681 |
| Arabinose isomeride* | O-4TMS | 1686 |
| 5-hydroxy, 1H-Indole | N,O-2TMS | 1712 |
| Ribitol isomeride* | O-5TMS | 1751 |
| Aconitate | O-3TMS | 1759 |
| Glutamine | N,O-TMS | 1784 |
| Glycerol-3-phosphate | O-4TMS | 1786 |
| Azelate | O-2TMS | 1800 |
| Hypoxanthine | N,O-2TMS | 1815 |
| Ornithine | N,O-4TMS | 1835 |
| Citrate | O-4TMS | 1843 |
| Glucose | MEOX, O-5TMS | 1925, 1954 |
| Histidine | N,O-3TMS | 1939 |
| Tyrosine | N,O-3TMS | 1961 |
| Gluconate | MEOX, O-5TMS | 2035 |
| Palmitic acid | O-TMS | 2048 |
| 3-Indolepropionic acid | N,O-2TMS | 2087 |
| Myo-inositol | O-6TMS | 2127 |
| Urate | N,O-4TMS | 2129 |
| Linoleic acid | O-TMS | 2213 |
| Oleic acid | O-TMS | 2218 |
| Tryptophan | N,O-3TMS | 2241 |
| Stearic acid | O-TMS | 2243 |
| Cystine | N,O-4TMS | 2318 |
| Arachidonic acid | O-TMS | 2362 |
| Pseudouridine | O-5TMS | 2364 |
| myo-Inositol-1-phosphate | O-7TMS | 2467 |
| Docosahexaenoic acid | O-TMS | 2564 |
| Sucrose | O-8TMS | 2704 |
| gamma-Tocopherol | O-TMS | 3012 |
| Adenosine-5-monophosphate | N, O-5TMS | 3114 |
| alpha-Tocopherol | O-TMS | 3166 |
| Cholesterol | O-TMS | 3185 |
| Campesterol | O-TMS | 3288 |
| Cholan-24-oic acid | O-3TMS | 3346 |
| beta-Sitosterol | O-TMS | 3375 |

- Compounds were not ambitious identified because of isomeric compounds of them.

Altogether, 72 compounds were identified in CML patients. Compounds are identified as TMS, trimethylsilylated, and MEOX, methoxymated derivatives. Identification was confirmed by comparing the mass spectra and retention indices with the authentic references standards and those available in the National Institute of Standards and Technology (NIST) library 2.0 (2005), an in-house mass spectra library database established by the Umeå Plant Science Center, and key lab of drug metabolism and pharmacokinetics, China Pharmaceutical Univers
