# Supplementary material for: Chronic Myeloid Leukemia Patients Sensitive and Resistant to Imatinib Treatment Show Different Metabolic Responses
Source: PLoS One. 2010 Oct 8;5(10):e13186. doi: 10.1371/journal.pone.0013186 (PMC2951899; doi:10.1371/journal.pone.0013186)
Supplement: Table S2 — The relative abundance of the identified compounds/peaks differentiating RCML BC from RCML CP patients. (0.06 MB DOC) [file pone.0013186.s005.doc]

**Supporting information,**

**Table S2：**

The relative abundance of the identified compounds/peaks differentiating RCML BC from RCML CP patients

|  | RCML BC | | | RCML CP | | | Folder change | ANOVA | Percent change |
| --- | --- | --- | --- | --- | --- | --- | --- | --- | --- |
| Identified Compounds | Mean | ± | SD | Mean | ± | SD | CP/BC | *p* | % |
| Oleate | 4.47E+06 | ± | 2272051 | 1.46E+07 | ± | 5836340 | 0.306 | 0.015 | -69.4 |
| Beta-D-Methylglucopyranoside | 4.02E+06 | ± | 1459828 | 1.09E+07 | ± | 4238858 | 0.369 | 0.020 | -63.1 |
| Palmitate | 4.44E+06 | ± | 1951023 | 1.05E+07 | ± | 2775245 | 0.422 | 0.004 | -57.8 |
| Arabinose | 1.15E+05 | ± | 45426 | 2.68E+05 | ± | 84159 | 0.431 | 0.011 | -56.9 |
| Linoleate | 3.90E+06 | ± | 1795301 | 8.28E+06 | ± | 2943088 | 0.471 | 0.025 | -52.9 |
| CPU_ DB5_RI2296_P | 1.72E+05 | ± | 64339 | 3.39E+05 | ± | 115881 | 0.507 | 0.028 | -49.3 |
| Pyroglutamate | 8.47E+06 | ± | 2063557 | 1.60E+07 | ± | 4117740 | 0.530 | 0.012 | -47.0 |
| Stearate | 2.52E+06 | ± | 729376 | 4.43E+06 | ± | 1113033 | 0.568 | 0.014 | -43.2 |
| Hydroxylamine | 1.52E+06 | ± | 529898 | 2.54E+06 | ± | 682844 | 0.600 | 0.049 | -40.0 |
| CPU_ DB5_RI1745_P | 1.70E+05 | ± | 62100 | 2.59E+05 | ± | 64922 | 0.655 | 0.038 | -34.5 |
| CPU_ DB5_RI2540_P | 4.42E+05 | ± | 188929 | 6.03E+05 | ± | 29909 | 0.733 | 0.047 | -26.7 |
| Urate | 1.11E+07 | ± | 3666265 | 1.51E+07 | ± | 2171126 | 0.735 | 0.031 | -26.5 |
| Urate* | 145.1 | ± | 33.7 | 257.0 | ± | 51.5 | 0.565 | 0.026 | -43.5 |
| 5-Hydroxy, 1H-Indole | 2.49E+05 | ± | 90140 | 3.35E+05 | ± | 23669 | 0.742 | 0.031 | -25.8 |
| Glycine | 2.97E+06 | ± | 952657 | 4.00E+06 | ± | 504938 | 0.743 | 0.028 | -25.7 |
| Fumarate | 3.47E+05 | ± | 54953 | 4.58E+05 | ± | 79303 | 0.758 | 0.032 | -24.2 |
| Lysine | 2.71E+05 | ± | 103381 | 1.39E+05 | ± | 95519 | 1.946 | 0.043 | 94.6 |

All peaks areas were normalized by the stable isotope internal standard, [^13^C_2_]-myristic acid; CPU_ DB5_RI*x*_P: Unidentified compounds in plasma samples detected with DB-5 capillary column in GC/TOFMS, China Pharmaceutical university (CPU); RI, retention index; *x*, retention time index value; P, plasma sample..

***,** clinic measurement results.
