# Supplementary material for: Chronic Myeloid Leukemia Patients Sensitive and Resistant to Imatinib Treatment Show Different Metabolic Responses
Source: PLoS One. 2010 Oct 8;5(10):e13186. doi: 10.1371/journal.pone.0013186 (PMC2951899; doi:10.1371/journal.pone.0013186)
Supplement: Table S3 — Primer pairs used for PCR. (0.04 MB DOC) [file pone.0013186.s006.doc]

**Supporting information,**

**Table S3 Primer pairs used for PCR**

| **Primer name** | **Sequence,5'-3'** | **Positions*** | **Ampllcon** |
| --- | --- | --- | --- |
|  |  |  | **(length)** |
| F-ABL1-A | GGA AAA GTA CTT GGG GAC CA | ABL(nt 455-474) | A( 555 bp) |
| R-ABL1-A | CAC CCT CCC TTC GTA TCT CA | ABL(nt 990-1009) |  |
| F-ABL1-B | TAC GAA GGG AGG GTG TAC CA | ABL(nt 995-1014) | B( 553bp) |
| R-ABL1-B | TGG CTG ACG AGA TCT GAG TG | ABL(nt 1528-1547) |  |
| F-ABL1-C | GCT GTA CAT GGC CAC TCA GA | ABL(nt 1516-1535) | C( 554bp) |
| R-ABL1-C | CCT GCA GCA AGG TAC TCA CA | ABL(nt 2050-2069) |  |
| F-ABL1-D | GTG GGG CTG TGA GTA CCT TG | ABL(nt 2043-2062) | D( 592bp) |
| R-ABL1-D | AAG CGC TTG CTG GAG CTG | ABL(nt 2617-2634) |  |
| F-ABL1-E | CCC CAC CTG TGG AAG AAG T | ABL(nt 2546-2564) | E( 632bp) |
| R-ABL1-E | CTG GAC AAT TTC CCC TTG TC | ABL(nt 3158-3177) |  |
| F-ABL1-F | GGC ACA AGC ACT CCT CTG A | ABL(nt 3126-3144) | F( 417bp) |
| R-ABL1-F | GGG TTT TCC GAA GAG ACA CTC | ABL(nt 3522-3542) |  |
| F-ABL1-G | CCT CTC ATA TCA ACC CGA GTG | ABL(nt 3506-3526) | G( 408bp) |
| R-ABL1-G | CTG ACA CCT GAC CCC TGA CT | ABL(nt 3506-3526) |  |

* Primer positions are according to GenBank Sequence from NM_007313 variant b, mRNA (for ABL1).
